# Microwave-Assisted One Pot Three-Component Synthesis of Novel Bioactive Thiazolyl-Pyridazinediones as Potential Antimicrobial Agents against Antibiotic-Resistant Bacteria

**DOI:** 10.3390/molecules26144260

**Published:** 2021-07-13

**Authors:** Saraa Abu-Melha, Sobhi M. Gomha, Amr S. Abouzied, Mastoura M. Edrees, Ahmed S. Abo Dena, Zeinab A. Muhammad

**Affiliations:** 1 Department of Chemistry, Faculty of Science, King Khalid University, Abha 61413, Saudi Arabia; sabomlba@kku.edu.sa (S.A.-M.); mstorh@kku.edu.sa (M.M.E.); 2 Chemistry Department, Faculty of Science, Cairo University, Giza 12613, Egypt; 3 Chemistry Department, Faculty of Science, Islamic University in Almadinah Almonawara, Almadinah Almonawara 42351, Saudi Arabia; 4 Pharmaceutical Chemistry Department, College of Pharmacy, University of Hail, Hail 81442, Saudi Arabia; amrabou.zied@yahoo.com; 5 Pharmaceutical Chemistry Department, National Organization for Drug Control and Research (NODCAR), Giza 12311, Egypt; ahmed_said5899@yahoo.com (A.S.A.D.); zeinab.a.muhammad@gmail.com (Z.A.M.); 6 Faculty of Oral and Dental Medicine, Future University in Egypt (FUE), New Cairo 11835, Egypt

**Keywords:** thiazolyl-pyridazinedione, hydrazonoyl chlorides, microwave irradiation, antibiotic-resistant bacteria, molecular docking

## Abstract

Pyridazine and thiazole derivatives have various biological activities such as antimicrobial, analgesic, anticancer, anticonvulsant, antitubercular and other anticipated biological properties. Chitosan can be used as heterogeneous phase transfer basic biocatalyst in heterocyclic syntheses. Novel 1-thiazolyl-pyridazinedione derivatives were prepared via multicomponent synthesis under microwave irradiation as ecofriendly energy source and using the eco-friendly naturally occurring chitosan basic catalyst with high/efficient yields and short reaction time. All the prepared compounds were fully characterized by spectroscopic methods, and their in vitro biological activities were investigated. The obtained results were compared with those of standard antibacterial/antifungal agents. DFT calculations and molecular docking studies were used to investigate the electronic properties and molecular interactions with specific microbial receptors.

## 1. Introduction

In recent years, a substantial number of pyridazine derivatives containing different moieties and/or substituents have demonstrated anti-inflammatory/analgesic, antipyretics, antiplatelet, anticancer, antidiabetic, antihypertensive, antidepressant/anxiolytic, anticonvulsant, antifungal, antibacterial, antitubercular, anti-bronchial asthma, antiallergic and other anticipated biological properties [1,2,3,4,5,6,7,8]. Moreover, thiazoles are considered an important class of heterocyclic compounds, found in many potent biologically active molecules such as sulfathiazole (an antimicrobial drug), Ritonavir (an antiretroviral drug), Abafungin (an antifungal drug) and Tiazofurin (an antineoplastic drug).

Continuously over the years, it has been noticed that interesting biological activities [9,10] were associated with thiazole derivatives. Recently, thiazoles have had a wide range of applications in drug development for the treatment of allergies, inflammation, schizophrenia, bacterial diseases, hypnotics and more recently for the treatment of pain, as fibrinogen receptor antagonists with antithrombotic activity and as new inhibitors of bacterial DNA gyrase B [11,12,13,14,15,16].

Multi-component reactions (MCRs) are one-pot processes with at least three components to form a single product, which incorporates most or even all of the starting materials [17,18,19,20]. The huge interest in such MCRs during the last years has been oriented towards developing combinatorial chemistry procedures, because of their high efficiency and convenience in comparison to multistage procedures. Additionally, the utility of MCRs under microwave irradiation (MWI) in the synthesis of heterocyclic compounds enhanced reaction rates and improved regioselectivity [21,22,23,24].

Chitosan, a biocompatible and biodegradable naturally occurring polysaccharide, is a copolymer containing both glucosamine and N-acetylglucosamine units. It can be used as a heterogeneous phase transfer basic biocatalyst in heterocyclic syntheses, such as enantioselective syntheses of asymmetric products with chiral center(s) [25,26], Michael addition reactions [27,28,29], as well as transition metal support for the preparation of heterogeneous catalysts [30]. The presence of amino groups is responsible for the basic nature of chitosan. Keeping this in mind, in continuation of our previously reported works on the synthesis of new biologically active agents [31,32,33,34,35,36,37], we present herein an efficient synthesis of novel 1-thiazolyl-pyridazinedione derivatives as antimicrobial agents, which have not been reported *hitherto* in a multicomponent synthesis under MWI as an ecofriendly energy source and using the eco-friendly naturally occurring chitosan catalyst.

## 2. Results and Discussion

### 2.1. Synthesis

**Scheme 1:**
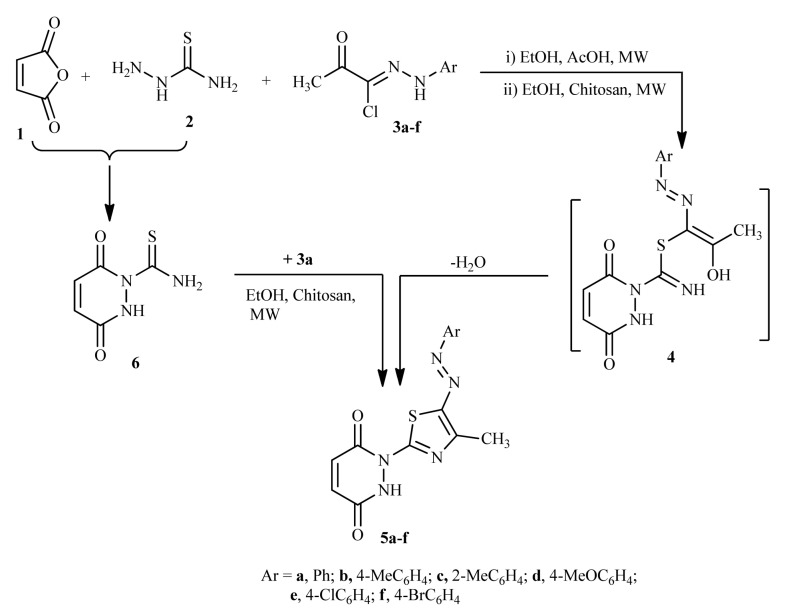
Synthesis of arylazothiazole derivatives **5a–f**.

In continuation of our previous work to synthesize bioactive heterocyclic compounds under mild conditions [38,39,40,41,42,43], we wish to report herein mild and efficient procedures for the synthesis of some novel 1-thiazolyl-pyridazinedione derivatives via the three-component reaction of maleic anhydride **1**, thiosemicarbazide **2** and the appropriate 2-oxo-N-arylpropanehydrazonoyl chlorides **3a–f** in ethanol in the presence of chitosan under MWI at 500 W and 150 °C for 4–8 min. as monitored by TLC (Scheme 1).

The structure of **5a–f** was confirmed by their spectral data (IR, MS and ^1^H-NMR), elemental analyses and alternative synthetic routes. For example, the ^1^H NMR spectra of compounds **5a–f** exhibited singlet signals at *δ* ~2.56 ppm (CH_3_) and one D_2_O exchangeable peaks at *δ* ~10.71 ppm corresponding to NH-phenyl, in addition to the expected signals for the aromatic protons and the two doublet signals of the CH=CH protons. The IR spectra of product **6** revealed in each case three absorption bands in the regions υ ~1654, 1668 and 3435 cm^−1^ due to the two carbonyl groups and NH group. The mass spectra of products **5a–f** revealed a molecular ion peak for each one, which is consistent with their respective molecular weights.

In the light of the foregoing results, the mechanism outlined in (Scheme 1) seems to be the most plausible pathway for the formation of compounds **5a–f** from the reaction of the **1** + **2** + **3**. The reaction involves initial formation of thiohydrazonate intermediate **4** via *S*-alkylation, with removal of HCl, which underwent dehydrative cyclization to afford the final product **5**.

Compound **5a** was alternatively synthesized by reacting carbothioamide **6** (prepared separately via condensation of maleic anhydride **1** and thiosemicarbazide **2**) with 2-oxo-N-phenylpropanehydrazonoyl chloride (**3a**) in ethanol containing catalytic amount of chitosan under MWI (Scheme 1). The obtained product was found to be identical with **5a** in all respects (TLC, mp. and IR spectrum), which affords further evidence to all structures **5a–f**.

**Scheme 2:**
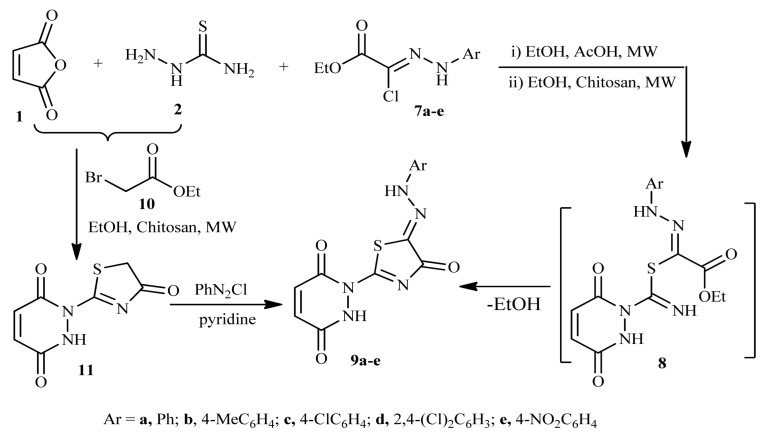
Synthesis of arylhydrazothiazolone derivatives **9a–e**.

In an identical way, when the three-component reaction of maleic anhydride **1**, thiosemicarbazide **2** and the appropriate ethyl (*N*-arylhydrazono)-chloroacetates **7a–e** under the same reaction condition, it yielded in each case a single product, namely, 1-(4-oxo-5-(2-arylhydrazono)-4,5-dihydrothiazol-2-yl)-1,2-dihydropyridazine-3,6-diones **9a–e** (Scheme 2).

The structure of compounds **9a–e** was proved based on spectral data, elemental analyses and chemical transformations. The spectroscopic information confirmed the reaction product **9** via intermediate **8** with elimination of EtOH molecule (Scheme 2).

Coupling of thiazolone **11** (prepared separately from reaction of carbothioamide derivative **6** with ethyl bromoacetate **10** in ethanol/chitosan under reflux) with PhN_2_Cl in pyridine yielded a product was found to be identical to **9a** in all regards (mp., TLC and IR spectrum), providing an additional evidence to all **9a–e** structures.

**Scheme 3:**
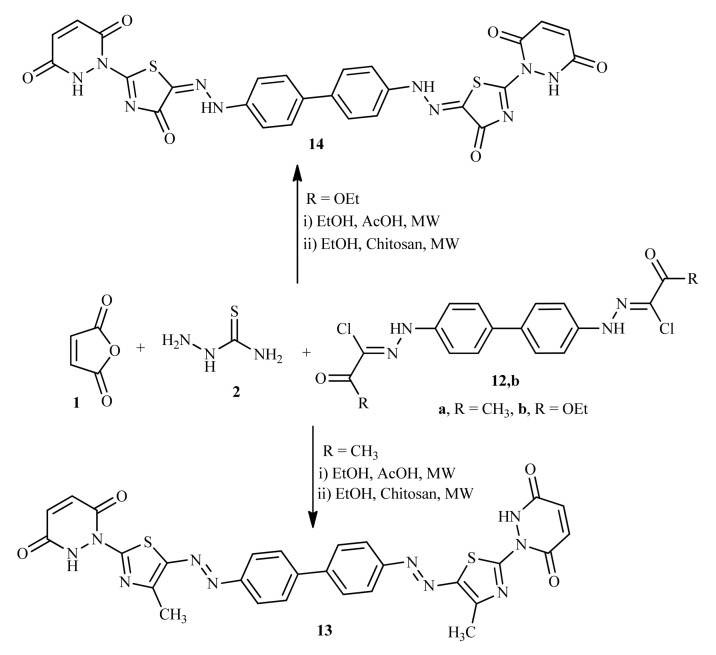
Synthesis of *bis*-thiazole derivatives **13** and **14**.

From literature reports [44,45,46,47] we found that compounds bearing more than one thiazole ring unit also exhibit good biological activities. For example, Myxothiazol is an inhibitor of the mitochondrial cytochrome bc1 complex and Bleomycin is an anticancer agent, containing 2,4′-*bis* thiazole system. From the above findings, we thought it is useful to synthesize a heterocyclic ring system carrying *bis*-thiazole moiety associated with pyridazine ring. This aim was achieved via the reaction of *bis*-hydrazonoyl chlorides **12a** and **12b** with two moles of maleimide **1** and two moles of thiosemicarbazide **2** under MWI in presence of chitosan to afford the respective *bis*-thiazoles **13** and **14** in a good yield (Scheme 3). The structure of compounds **13** and **14** was proven based on spectral data and elemental analyses (Experimental part).

### 2.2. XTT Assay Results

The minimum inhibitory concentration (MIC) of the tested compounds on cell metabolism/viability of *S. aureus*, *P. aeruginosa* and *C. albicans* was determined using XTT assay compared to the standard counterparts (vancomycin and amphotericin B).

The results presented in Table 1 depict that most of the investigated compounds have higher activities towards bacterial strains than fungal ones. Compound **9d** has a low MIC and acts against all resistant bacterial (*P. aeruginosa* and *S. aureus*, MIC: 0.42 and 1.84 g/mL, respectively) and fungal (*Candida albicans*, MIC: 2.17 g/mL) strains, indicating that it has a significant antibacterial and antifungal activity. Compounds **5a**, **5b**, **5c**, **5e**, **5f**, **9b**, **9c**, and **13**, on the other hand, show no effect on the azole-resistant *C. albicans* ATCC10231 fungus. The majority of the studied molecules demonstrate different degrees of activity towards the resistant *S. aureus* (MRSA) TCC-BAA-1720. Compounds **5d**, **9b**, and **14** appeared to be the most effective. Compound **5d** was more effective than the reference drug vancomycin against the sensitive *Pseudomonas aeruginosa* ATCC 10145 and resistant *Pseudomonas aeruginosa* ATCC BAA-2108. Compounds **5c**, **5d**, **9b**, **9c** and **13** showed no activity against the resistant *Pseudomonas aeruginosa* ATCC BAA-2108. In addition, compound **14** shows a good antimicrobial activity against *S. aureus* and *P. aeruginosa* (MIC: 1.13 and 1.49 µg/mL, respectively). In order to correlate the in silico results with those of the experimental antibacterial testing, SAP and FabI receptors were chosen for docking with the tested compounds.

### 2.3. Molecular Modeling

**Figure 1:**
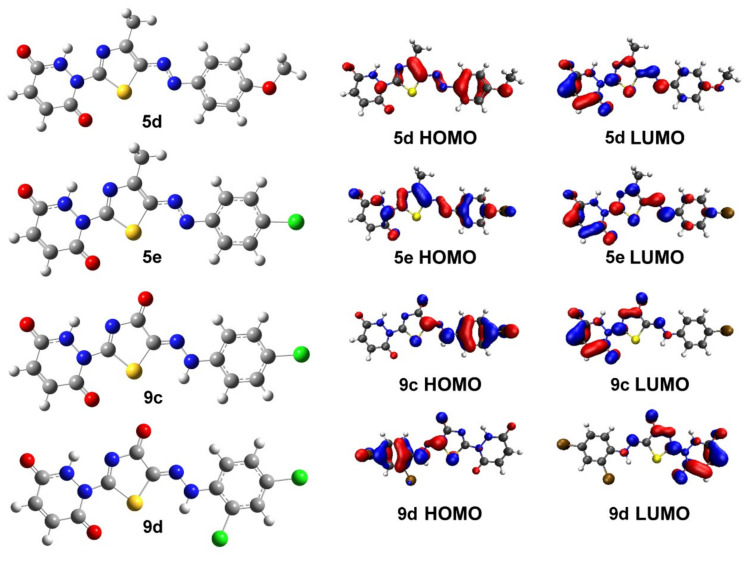
Optimized molecular geometries and molecular orbitals of candidate molecules with the highest antimicrobial properties.

At the B3LYP/6-311G level of theory, the geometries of the synthesized molecules that demonstrated the greatest biological activity in the XTT experiments (**5d**, **5e**, **9c**, and **9d**) were investigated (Figure 1). The findings revealed that the molecules under investigation are nearly planar. The highest occupied molecular orbitals (HOMO) are noticed on the substituted phenyl and thiazole rings in all of the investigated compounds, whereas the lowest unoccupied molecular orbitals (LUMO) are found on the pyridazine-3,6-dione rings. Molecular orbital analysis can give information about the reactivity and excitability of the studied molecules. From HOMO/LUMO analysis, it can be concluded that molecules with narrow energy gaps (e.g., **5d** and **9c**) may show better reactivity/excitability than those having wide energy gaps (e.g., **5e** and **9d**).

The quantum mechanical descriptors of the picked molecules are summarized in Table 2. The energy gaps between HOMO and LUMO were discovered to be in the range of 2.87 to 3.06 eV, with **5d** having the smallest energy gap.

Molecular docking was used to study the ligand-receptor interactions that may result in the obtained biological activities of the studied molecules. Thiazole derivatives have been reported to exhibit strong antibacterial activity against *Staphylococcus aureus* and *Candida albicans*. As a result, the studied candidate chemicals have strong antibacterial activity against these two pathogens. Furthermore, antibacterial activity against *Pseudomonas aeruginosa* was established by the substances under investigation. Accordingly, we chose the most appropriate receptors from the organisms mentioned above for molecular docking investigations.

During disseminated/mucosal infections of *Candida albicans*, secreted aspartic proteinase (SAP) plays a key function as a virulence factor. This receptor is assumed to be involved in the fungus’ attachment and invasion, and so plays a role in its pathogenicity. As a result, SAPs may be useful as pharmacological target receptors for candidiasis treatment [48].

*Staphylococcus aureus* is a common Gram-positive bacterium that can cause wound infections and staphylococcal scalded skin syndrome (a cutaneous reaction to a staphylococcal exotoxin absorbed into the circulation) [49]. One of the essential components of the FAS II system (a group of fatty acid synthases used by most of bacteria and plants to catalyze fatty acid synthesis) is enoyl-[acyl-carrier-protein] reductase (FabI). Other bacteria, such as *Pseudomonas aeruginosa*, require this enzyme as well. In order to correlate the in silico results with those obtained from the experimental antibacterial tests, SAP and FabI were chosen for docking with the compounds of interest.

**Figure 2:**
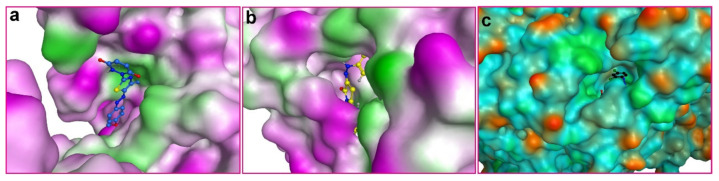
Structure of receptor molecules with the best fitting ligand molecules from the investigated set of ligands. (**a**) SAP2 of *C. albicans*/**5d**, (**b**) FabI of *S. aureus*/**9c** and (**c**) FabI of *P. aeruginosa*/**9d**.

**Figure 3:**
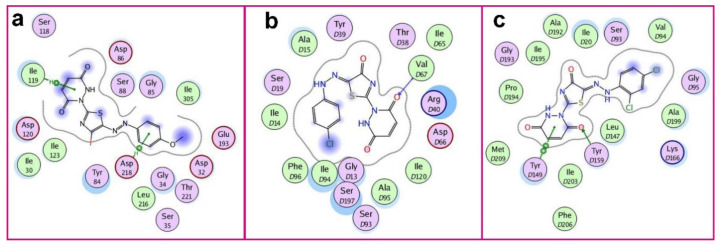
Ligand interaction diagrams for the best fitting ligand molecules showing the amino acid residues of the receptor active site and their interaction with the ligands. (**a**) SAP2 of *C. albicans*/**5d**, (**b**) FabI of *S. aureus*/**9c** and (**c**) FabI of *P. aeruginosa*/**9d**.

Figure 2 and Figure 3 depict the layouts of the receptors under investigation. and their interactions with the studied ligands. Molecular docking revealed that compounds **5d**, **9c**, and **9d** are the best ligands for SAP2 of *Candida albicans*, FabI of *S. aureus*, and FabI of *P. aeruginosa*, respectively. The calculated docking scores were found to be −11.35, −11.30 and −11.36 kcal/mol for SAP2 of *C. albicans*/**5d**, FabI of *S. aureus*/**9c** and FabI of *P. aeruginosa*/**9d**, respectively.

The in silico studies revealed that the of interaction of ligand **5d** with SAP2 of *C. albicans* occurs via the hydrogen-aryl interaction between the aryl group of the ligand and the Asp 218 residue, and between the quinoid ring of the ligand and the Ile 119 amino acid. Whereas ligand **9c** interacts with FabI of *S. aureus* through hydrogen bonding with Val D67 amino acid residue. In addition, compound **9d** interacts with FabI of *P. aeruginosa* by aryl interaction with Tyr D149 and via the formation of a hydrogen bond with Tyr D159.

By comparing the results of in vitro XTT assay with those of the molecular docking study, it can be obviously noticed that there is an excellent agreement between them. For instance, compound **5d,** which shows the best docking score with SAP2 of *C. albicans*, is active against both *C. albicans* and *S. aureus* as shown in Table 1. In addition, ligand **9c,** which demonstrated the best binding to Fab I of S. aureus, was found to be inactive against all microorganisms but *S. aureus*, as indicated from the XTT assay; thus, confirming the accuracy of the docking studies. Furthermore, compound **9d** interestingly demonstrated a better antimicrobial activity against *P. aeruginosa* (MIC = 0.24 µg/mL) than the standard molecule, vancomycin (MIC = 0.49 µg/mL). This agrees with the activity predicted from docking which revealed that ligand **10d** has the best docking score (−11.36 kcal/mol) amongst all the theoretically studied ligands.

## 3. Materials and Methods

### 3.1. General Experimental Procedures

Melting points were measured with an Electrothermal IA 9000 series digital melting point apparatus. IR spectra were recorded in potassium bromide discs on PyeUnicamSP 3300 and Shimadzu FTIR 8101 PC infrared spectrophotometers. NMR spectra were recorded on a Varian Mercury VX-300 NMR spectrometer operating at 300 MHz (^1^HNMR) and run in deuterated dimethylsulfoxide (DMSO-*d*_6_). Chemical shifts were related to that of the solvent. Mass spectra were recorded on a Shimadzu GCeMS-QP1000 EX mass spectrometer at 70 eV. Elemental analyzes were measured by using a German made ElementarVario LIII CHNS analyzer. Irradiation was done in an ultrasonicator, (Electric supply: 230 v, A.C. 50 Hz, 1phase; Ultrasonic frequency: 36 KHz; Ultrasonic power: 100 W). Maleic anhydride **1**, thiosemicarbazide **2**, chitosan, aniline and pyridine were purchased from Sigma Aldrich Kingdom of Saudi Arabia and were used without further purification. Hydrazonoyl halides **3a–f, 7a–e** and *bis*-hydrazonoyl halides **12a,b** were prepared according to the reported methods [50,51].

### 3.2. Synthesis of Thiazole Derivatives ***5a–f***, and ***9a–e***

An equivalent amount of glacial acetic acid (0.5 mL) was added to a solution of maleic anhydride **1** (0.98 g, 1 mmol), thiosemicarbazide **2** (0.92 g, 1 mmol) in ethanol (20 mL). The reaction mixture was heated in microwave oven at 500 W and 150 °C for 2 min. Then, the appropriate hydrazonoyl halides **3a–f** or **7a–e** and chitosan (0.1 g) were added, the reaction mixture was further heated in microwave oven at 500 W and 150 °C until all the starting material was consumed (4–8 min. as monitored by TLC). The hot solution was filtered to remove chitosan and excess solvent was removed under reduced pressure. The reaction mixture was triturated with methanol and the product separated was filtered, washed with methanol, dried and recrystallized from EtOH or DMF to give products **5a–f** and **9a–e,** respectively. The analytical and spectral data of the products **5a–f** and **9a–e** are listed below.

#### 3.2.1. 1-(4-Methyl-5-(phenyldiazenyl)thiazol-2-yl)-1,2-dihydropyridazine-3,6-dione) (**5a**)

Red fine crystals; m.p. 184–186 °C (DMF). Anal. Calcd. for C_14_H_11_N_5_O_2_S (313.33): C, 53.67; H, 3.54; N, 22.35. Found C, 53.55; H, 3.35; N, 22.14%. MS *m/z* (%) 313 (M^+^, 12), 250 (7), 233 (18), 149 (23), 133 (30), 128 (41), 113 (60), 98 (39), 73 (100), 65 (41), 55 (91); ^1^H-NMR (DMSO-*d*_6_): *δ* 2.56 (s, 3H, CH_3_), 6.24 (d, *J* = 12 Hz, 1H, CH=CH), 6.66 (d, *J* = 12 Hz, 1H, CH=CH), 7.01–7.56 (m, 5H, Ar-H), 10.62 (br s, 1H, NH) ppm; ^13^C-NMR (DMSO-*d*_6_): δ 19.5 (CH_3_), 109.9, 114.1, 120.7, 128.6, 129.9, 131.5, 132.3, 139.3, 143.4 (Ar-C and C=N), 156.7, 159.4 (2C=O) ppm; IR (KBr): *v* 3435 (NH), 3049, 2926 (C-H), 1668, 1654 (2C=O) cm^−1^.

#### 3.2.2. 1-(4-Methyl-5-(p-tolyldiazenyl)thiazol-2-yl)-1,2-dihydropyridazine-3,6-dione) (**5b**)

Dark red fine crystals; m.p. 171–173 °C (EtOH); Anal. Calcd. for C_15_H_13_N_5_O_2_S (327.36): C, 55.04; H, 4.00; N, 21.39. Found C, 55.35; H, 3.70; N, 21.18%. MS *m/z* (%) 327 (M^+^, 5), 270 (14), 199 (16), 159 (77), 133 (9), 106 (76), 91 (100), 77 (43), 57 (33); ^1^H-NMR (DMSO-*d*_6_): *δ* 2.36 (s, 3H, CH_3_), 2.56 (s, 3H, CH_3_), 6.23 (d, *J* = 12 Hz, 1H, CH=CH), 6.52 (d, *J* = 12 Hz, 1H, CH=CH), 7.15–7.31 (m, 4H, Ar-H), 10.66 (br s, 1H, NH) ppm; IR (KBr): *v* 3429 (NH), 3027, 2921 (C-H), 1690, 1654 (2C=O) cm^−1^.

#### 3.2.3. 1-(4-Methyl-5-(m-tolyldiazenyl)thiazol-2-yl)-1,2-dihydropyridazine-3,6-dione) (**5c**)

Dark red fine crystals; m.p. 185–187 °C. Anal. Calcd. for C_15_H_13_N_5_O_2_S (327.36): C, 55.04; H, 4.00; N, 21.39. Found C, 55.25; H, 3.79; N, 21.17%. MS *m/z* (%) 327 (M^+^, 8), 222 (48), 129 (21), 91 (58), 77 (36), 63 (100). ^1^H-NMR (DMSO-*d*_6_): *δ* 2.34 (s, 3H, CH_3_), 2.45 (s, 3H, CH_3_), 6.23 (d, *J* = 12 Hz, 1H, CH=CH), 6.48 (d, *J* = 12 Hz, 1H, CH=CH), 7.06–7.64 (m, 4H, Ar-H), 10.93 (br s, 1H, NH); IR (KBr): *v* 3433 (NH), 3011, 2923 (C-H), 1683, 1669 (2C=O) cm^−1^.

#### 3.2.4. 1-(5-((4-Methoxyphenyl)diazenyl)-4-methylthiazol-2-yl)-1,2-dihydropyridazine-3,6-dione (**5d**)

Dark red fine crystals; m.p. 170–172 °C (DMF). Anal. Calcd. for C_15_H_13_N_5_O_3_S (343.07): C, 52.47; H, 3.82; N, 20.40. Found C, 52.48; H, 3.65; N, 20.23%. MS *m/z* (%) 343 (M^+^, 3), 313 (6), 199 (5), 129 (11), 108 (15), 97 (27), 73 (40), 57 (100). ^1^H-NMR (DMSO-*d*_6_): δ 2.66 (s, 3H, CH_3_), 3.79 (s, 3H, OCH_3_), 6.23 (d, *J* = 12 Hz, 1H, CH=CH), 6.62 (d, *J* = 12 Hz, 1H, CH=CH), 7.03–7.86 (m, 4H, Ar-H), 10.87 (br s, 1H, NH) ppm; ^13^C-NMR (DMSO-*d*_6_): δ 19.1 (CH_3_), 55.3 (OCH_3_), 110.3, 113.6, 114.5, 126.9, 127.2, 128.7, 132.7, 149.6, 153.7 (Ar-C), 157.1, 159.1 (2C=O) ppm; IR (KBr): *v* 3423 (NH), 3022, 2924 (C-H), 1676, 1659 (2C=O) cm^−1^.

#### 3.2.5. 1-(5-((4-Chlorophenyl)diazenyl)-4-methylthiazol-2-yl)-1,2-dihydropyridazine-3,6-dione (**5e**)

Dark red fine crystals; m.p. 195–197 °C (DMF). Anal. Calcd. for C_14_H_10_ClN_5_O_2_S (397.04): C, 48.35; H, 2.90; N, 20.14. Found C, 48.75; H, 2.74; N, 19.98%; MS *m/z* (%) 397 (M^+^, 12), 283 (4), 267 (22), 185 (4), 152 (8), 129 (26), 111 (60), 99 (66), 86 (61), 57 (100); ^1^H-NMR (DMSO-*d*_6_): *δ* 2.56 (s, 3H, CH_3_), 6.27 (d, *J* = 12 Hz, 1H, CH=CH), 6.64 (d, *J* = 12 Hz, 1H, CH=CH), 7.26–8.12 (m, 4H, Ar-H), 10.84 (br s, 1H, NH) ppm; IR (KBr): *v* 3433 (NH), 3042, 2925 (C-H), 1671, 1657 (2C=O) cm^−1^.

#### 3.2.6. 1-(5-((4-Bromophenyl)diazenyl)-4-methylthiazol-2-yl)-1,2-dihydropyridazine-3,6-dione (**5f**)

Brown fine crystals; m.p. 207–209 °C (DMF). Anal. Calcd. for C_14_H_10_BrN_5_O_2_S (392.23): C, 42.87; H, 2.57; N, 17.86. Found C, 43.21; H, 2.25; N, 17.55%. MS *m/z* (%) 392 (M^+^, 2), 325 (53), 274 (11), 171 (25), 129 (15), 91 (57), 86 (89), 73 (64), 57 (100); ^1^H-NMR (DMSO-*d*_6_): *δ* 2.43 (s, 3H, CH_3_), 6.12 (d, *J* = 12 Hz, 1H, CH=CH), 6.46 (d, *J* = 12 Hz, 1H, CH=CH), 7.39–8.20 (m, 4H, Ar-H), 11.23 (br s, 1H, NH) ppm; IR (KBr): *v* 3434 (NH), 3032, 2923 (C-H), 1685, 1660 (2C=O) cm^−1^.

#### 3.2.7. 1-(4-Oxo-5-(2-phenylhydrazineylidene)-4,5-dihydrothiazol-2-yl)-1,2-dihydropyridazine-3,6-dione (**9a**)

Yellow fine crystals; m.p. 161–163 °C (EtOH). Anal. Calcd. for C_13_H_9_N_5_O_3_S (315.31): C, 49.52; H, 2.88; N, 22.21. Found C, 49.70; H, 2.57; N, 21.88%. MS *m/z* (%) 315 (M^+^, 7), 307 (100), 279 (22), 150 (14), 104 (10), 92 (67), 77 (35), 65 (29); ^1^H-NMR (DMSO-*d*_6_): *δ* 6.20 (d, *J* = 12 Hz, 1H, CH=CH), 6.41 (d, *J* = 12 Hz, 1H, CH=CH), 7.04–7.82 (m, 5H, Ar-H), 10.75, 11.00 (2br s, 2H, 2NH) ppm; ^13^C-NMR (DMSO-*d*_6_): δ 110.3, 116.3, 117.9, 123.1, 129.0, 130.3, 131.5, 147.7 (Ar-C and C=N), 158.2, 161.7, 172.8 (3C=O) ppm; IR (KBr): *v* 3429, 3178 (2NH), 3040, 2975 (C-H), 1706, 1680, 1653 (3C=O) cm^−1^.

#### 3.2.8. 1-(4-Oxo-5-(2-(p-tolyl)hydrazineylidene)-4,5-dihydrothiazol-2-yl)-1,2-dihydropyridazine-3,6-dione (**9b**)

Yellow fine crystals; m.p. 154–156 °C (EtOH). Anal. Calcd. for C_14_H_11_N_5_O_3_S (329.33): C, 51.06; H, 3.37; N, 21.27. Found C, 51.35; H, 3.06; N, 21.03%. MS *m/z* (%) 329 (M^+^, 7), 263 (12), 155 (18), 125 (4), 111 (10), 101 (16), 97 (15), 86 (100), 58 (46); ^1^H-NMR (DMSO-*d*_6_): *δ* (s, 3H, CH_3_), *δ* 6.23 (d, *J* = 12 Hz, 1H, CH=CH), 6.45 (d, *J* = 12 Hz, 1H, CH=CH), 7.40–8.00 (m, 4H, Ar-H), 10.54, 10.79 (2 br s, 2H, 2NH); IR (KBr): *v* 3431, 3278 (2NH), 3030, 2979 (C-H), 1705, 1679, 1629 (3C=O) cm^−1^.

#### 3.2.9. 1-(5-(2-(4-Chlorophenyl)hydrazineylidene)-4-oxo-4,5-dihydrothiazol-2-yl)-1,2-dihydropyridazine-3,6-dione (**9c**)

Yellow fine crystals; m.p. 170–172 °C (EtOH). Anal. Calcd. for C_13_H_8_ClN_5_O_3_S (349.75): C, 44.64; H, 2.31; N, 20.02. Found C, 44.93; H, 2.01; N, 19.70%. MS *m/z* (%) 351 (M^+^+2, 2), 349 (M^+^, 7), 310 (5), 239 (5), 152 (10), 125 (36), 111 (31), 83 (39), 69 (58), 57 (100); ^1^H-NMR (DMSO-*d*_6_): *δ* 6.30 (d, *J* = 12 Hz, 1H, CH=CH), 6.54 (d, *J* = 12 Hz, 1H, CH=CH), 6.93–7.58 (m, 4H, Ar-H), 9.96, 12.68 (2 br s, 2H, 2NH) ppm; IR (KBr): *v* 3431, 3219 (2NH), 3039, 2989 (C-H), 1705, 1657, 1629 (3C=O) cm^−1^.

#### 3.2.10. 1-(5-(2-(2,4-Dichlorophenyl)hydrazineylidene)-4-oxo-4,5-dihydrothiazol-2-yl)-1,2-dihydropyridazine-3,6-dione (**9d**)

Yellow fine crystals; m.p. 149–151 °C (EtOH). Anal. Calcd. for C_13_H_7_Cl_2_N_5_O_3_S (382.19): C, 40.64; H, 1.84; N, 18.23. Found C, 40.93; H, 1.55; N, 18.00%. MS *m/z* (%) 382 (M^+^, 3), 375 (100), 349 (12), 347 (18), 218 (5), 160 (21), 133 (21), 112 (9), 82 (12); ^1^H-NMR (DMSO-*d*_6_): *δ* 6.30 (d, *J* = 12 Hz, 1H, CH=CH), 6.54 (d, *J* = 12 Hz, 1H, CH=CH), 6.93–7.58 (m, 3H, Ar-H), 9.96, 12.68 (2 br s, 2H, 2NH) ppm; IR (KBr): *v* 3383, 3219 (2NH), 3039, 2983 (C-H), 1696, 1657, 1641 (3C=O) cm^−1^.

#### 3.2.11. 1-(5-(2-(4-Nitrophenyl)hydrazineylidene)-4-oxo-4,5-dihydrothiazol-2-yl)-1,2-dihydropyridazine-3,6-dione (**9e**)

Yellow fine crystals; m.p. 159–161 °C (EtOH). Anal. Calcd. for C_13_H_8_N_6_O_5_S (360.30): C, 43.34; H, 2.24; N, 23.33. Found C, 43.42; H, 2.09; N, 23.15%; MS *m/z* (%) 360 (M^+^, 12), 328 (9), 259 (4), 221 (3), 180 (4), 152 (4), 129 (12), 113 (40), 97 (27), 87 (38), 71 (48), 59 (100); ^1^H-NMR (DMSO-*d*_6_): *δ* 2.26 (s, 3H, CH_3_), 6.27 (d, *J* = 12 Hz, 1H, CH=CH), 6.51 (d, *J* = 12 Hz, 1H, CH=CH), 6.98–7.97 (m, 4H, Ar-H), 10.54, 10.77 (2 br s, 2H, 2NH) ppm; IR (KBr): *v* 3426, 3178 (2NH), 3030, 2922 (C-H), 1703, 1649, 1632 (3C=O) cm^−1^.

### 3.3. Alternate Synthesis of Thiazole Derivative ***5a***

#### 3.3.1. Synthesis of 3,6-Dioxo-3,6-dihydropyridazine-1(2*H*)-carbothioamide (**6**)

An equivalent amount of glacial AcOH (0.5 mL) was added to an ethanolic solution of maleic anhydride **1** (0.098 g, 1 mmol) and thiosemicarbazide **2** (0.092 g, 1 mmol). The reaction mixture was heated in a microwave oven at 500 W and 150 °C for 2 min. as monitored by TLC. The formed product was recrystallized from ethanol to give pure derivative **6** as white solid; m.p. 232–234 °C. Anal. Calcd. for C_5_H_5_N_3_O_2_S (171.17): C, 35.08; H, 2.94; N, 24.55. Found C, 35.01; H, 2.84; N, 24.49%; MS *m/z* (%): 171 (M^+^), 132 (19), 107 (80), 87 (53), 57 (100); ^1^H-NMR (DMSO-*d*_6_): *δ* 6.19 (d, *J* = 12 Hz, 1H, CH=CH), 6.44 (d, *J* = 12 Hz, 1H, CH=CH), 9.30 (br s, 2H, NH_2_), 10.46 (br s, 1H, NH) ppm; IR (KBr): *v* 3387–3314, 3258 (NH_2_ and NH), 3149, 2963 (C-H), 1687, 1634 (2C=O) cm^−1^.

#### 3.3.2. Reaction of **6** with **3a**

Equimolar amounts of carbothioamide **7** (0.171 g, l mmol) and 2-oxo-N-phenylpropane hydrazonoyl chloride **3a** (0.196 g, mmol) in ethanol (15 mL) containing an equivalent amount of chitosan (0.1 g) was heated in a microwave oven at 500 W and 150 °C for 5 min. as monitored by TLC. The hot solution was filtered to remove chitosan and excess solvent was removed under reduced pressure, gave product identical in all respects (m.p., mixed m.p. and IR spectra) with compounds **5a**.

### 3.4. Alternate Synthesis of Thiazole Derivative ***9a***

#### 3.4.1. 1-(4-Oxo-4,5-dihydrothiazol-2-yl)-1,2-dihydropyridazine-3,6-dione (**11**)

To a solution of maleic anhydride **1** (0.098 g, 1 mmol), thiosemicarbazide **2** (0.092 g, 1 mmol), and ethyl 2-bromoacetate **10** (0.0165 g, 1 mmol) in ethanol (15 mL), an equivalent amount of chitosan (0.1 g) was added. The reaction mixture was heated in microwave oven at 500 W and 150 °C for 4 min. as monitored by TLC. The hot solution was filtered to remove chitosan and excess solvent was removed under reduced pressure. The reaction mixture was triturated with methanol and the product separated was filtered, washed with methanol, dried and recrystallized from ethanol to give thiazolone products **11** as yellow solid; m.p. 157–159 °C; Anal. Calcd. for C_7_H_5_N_3_O_3_S (211.01): C, 39.81; H, 2.39; N, 19.90. Found C, 40.21; H, 2.17; N, 19.64%. MS *m/z* (%) 211 (M^+^, 16), 149 (19), 117 (63), 92 (66), 57 (100); ^1^H-NMR (DMSO-*d*_6_): *δ* 3.82 (s, 2H, CH_2_), 6.22 (d, *J* = 12 Hz, 1H, CH=CH), 6.53 (d, *J* = 12 Hz, 1H, CH=CH), 10.25 (s, 1H, NH) ppm; IR (KBr): *v* 3438 (NH), 3168, 2987 (C-H), 1708, 1650, 1648 (3C=O) cm^−1^.

#### 3.4.2. Coupling of **11**

To a solution of each of compound **10** (0.211 g, 1 mmol) with sodium acetate trihydrate in ethanol (10 mL) was added benzenediazonium chloride solution, (prepared as usual by diazotizing aniline (1 mmol) in hydrochloric acid (1 mL, 6 M) with sodium nitrite (0.07 g, 1 mmol) in 10 mL water) portion wise with stirring and cooling. After complete addition, the reaction mixture was left for 12 h. in the refrigerator. The precipitate formed was collected by filtration, washed with water, dried and then recrystallized from EtOH to give the respective product identical in all respects with **9a**.

### 3.5. Synthesis of Bis-Thiazole ***13*** and Bis-Thiazolone ***14***

To a solution of maleic anhydride **1** (0.196 g, 2 mmol), thiosemicarbazide **2** (0.184 g, 2 mmol) in ethanol (20 mL), an equivalent amount of glacial acetic acid (1 mL) was added. The reaction mixture was heated in microwave oven at 500 W and 150 °C for 2 min. Then, the appropriate *bis*-hydrazonoyl halides **12a,b** (1 mmol for each) and chitosan (0.2 g) were added, the reaction mixture was further heated in microwave oven at 500 W and 150 °C until all the starting material was consumed (8 min as monitored by TLC). The hot solution was filtered to remove chitosan and excess solvent was removed under reduced pressure. The reaction mixture was triturated with methanol and the product separated was filtered, washed with methanol, dried and recrystallized from ethanol to give products **13** and **14,** respectively.

#### 3.5.1. 1,1′-(([1,1′-Biphenyl]-4,4′-diylbis(diazene-2,1-diyl))bis(4-methylthiazole-5,2-diyl))bis(1,2-dihydropyridazine-3,6-dione) (**13**)

Yellow fine crystals; m.p. 187–189 °C; Anal. Calcd. for C_28_H_20_N_10_O_4_S_2_ (624.11): C, 53.84; H, 3.23; N, 22.42. Found C, 54.04; H, 3.09; N, 22.27%; MS *m/z* (%) 624 (M^+^, 22), 373 (13), 341 (10), 299 (2), 271 (21), 112 (20), 98 (39), 86 (67), 69 (45), 54 (100); ^1^H-NMR (DMSO-*d*_6_): δ 2.58 (s, 6H, CH_3_), 6.27 (d, *J* = 12 Hz, 2H, CH=CH), 6.63 (d, *J* = 12 Hz, 2H, CH=CH), 7.43 (s, 8H, Ar-H), 10.64 (br s, 2H, 2NH) ppm; ^13^C-NMR (DMSO-*d*_6_): δ 19.7 (CH_3_), 109.5, 115.7, 120.7, 127.5, 133.2, 137.9, 142.1, 143.1, 152.1 (Ar-C and C=N), 156.8, 160.0 (2C=O) ppm; IR (KBr): v 3428 (NH), 2922 (C-H), 1699, 1659 (2C=O) cm^−1^.

#### 3.5.2. 1-(5-(2-(4′-(2-(2-(3,6-Dioxo-3,6-dihydropyridazin-1(2*H*)-yl)-4-oxothiazol-5(4*H*)-ylidene)hydrazinyl)-[1,1′-biphenyl]-4-yl)hydrazineylidene)-4-oxo-4,5-dihydrothiazol-2-yl)-1,2-dihydropyridazine-3,6-dione (**14**)

Yellow fine crystals; m.p. 198–200 °C; Anal. Calcd. for C_26_H_16_N_10_O_6_S_2_ (628.60): C, 49.68; H, 2.57; N, 22.28. Found C, 49.59; H, 2.48; N, 22.10%; MS *m/z* (%) 628 (M^+^, 4), 367 (31), 334 (24), 313 (19), 294 (49), 236 (25), 184 (63), 139 (66), 97 (36), 71 (49), 55 (100); ^1^H-NMR (DMSO-*d*_6_): *δ* 6.29 (d, *J* = 12 Hz, 2H, CH=CH), 6.53 (d, *J* = 12 Hz, 2H, CH=CH), 7.52 (m, 8H, Ar-H), 10.37, 10.79 (2 br s, 4H, 2NH) ppm; IR (KBr): *v* 3422, 3032 (2NH), 2978, 2930 (C-H), 1683, 1655, 1636 (3C=O) cm^−1^.

### 3.6. In Vitro XTT Assay

XTT assay, a non-radioactive colorimetric assay system, is usually used for measuring cell viability, proliferation and cytotoxicity through the measurement of cellular metabolic activity. This test depends on the reduction of a yellow tetrazolium salt (XTT dye) to an orange formazan dye by metabolically active cells. The minimal inhibitory concentration (MIC) values, which represent the lowest concentrations of samples or standard drugs (Vancomycine for bacteria and Amphotricine B for fungi) that completely inhibit the microbial growth. MICs were determined using the microdilution method. The bacterial inoculum was prepared, and the suspensions were adjusted to 10^6^ CFU/mL. The samples under investigation and the standard drugs were prepared in dimethyl sulfoxide (DMSO) and subsequent twofold dilutions were performed in a 96-well plate. Each well of the microplate included 40 μL of the growth medium (Brain Heart Infusion, BHI), 10 μL of the inoculum and 50 μL of the investigated compounds diluted to final concentrations of (1000–0.12 µg/mL), and DMSO was used as a negative control. The plates were incubated at 37 °C for 24 h. Thereafter, 40 μL of tetrazolium salt {2,3-bis[2-methyloxy-4-nitro-5-sulfophenyl]-2H-tetrazolium-5-car-boxanilide (XTT)} were added. The plates were incubated in dark for 1 h at 37 °C, after which colorimetric change in the XTT reduction assay was measured using a microtiter plate reader (Tecan Sunrise absorbance reader; Tecan UK, Reading, United Kingdom) at 492 nm. The MIC was detected as the lowest concentration capable of causing the largest color change compared to the negative control [52].

### 3.7. In Silico Studies

The electronic properties of the synthesized derivatives that demonstrated the best biological activities in the in vitro XTT assay were investigated with density functional theory calculations. The calculations were carried out with the aid of Gaussian 09 [53]. The geometry of the studied molecules was fully optimized using B3LYP/6-311G functional and the obtained molecular orbitals were visualized.

Molecular docking was used to investigate the interaction of the best biologically active molecules with the microbial receptors. We selected the most probable bacterial/fungal proteins that can be affected by the synthesized thiazole ligands based on the results previously reported in the literature. Molecular docking was carried out with the aid of the Molecular Operating Environment (MOE) 2014 software [54]. The geometry-optimized compounds that demonstrated the lowest MIC values in the XTT assay were selected and docked with the corresponding receptors. High-resolution 3D molecular structures of the receptors Secreted Aspartic Proteinase (SAP2; *C. albicans*; PDB ID: 1EAG), Enoyl-acyl Carrier Protein Reductase (fabI; *S. aureus*; PDB ID: 3GR6) and Enoyl-acyl Carrier Protein Reductase (FabI; *P. aerugiosa*; PDB ID: 4NR0) were obtained from the Protein Data Bank (PDB).

## 4. Conclusions

In summary, we have developed a new green methodology and synthesized several novel 1-thiazolylpyridazine derivatives by MWI in high, efficient yields and short reaction time. Additionally, the antimicrobial activities of the candidate lead molecules were tested against *S. aureus*, *P. aeruginosa* and *C. albicans* using the XTT assay and compounds with the highest activity in terms of MIC were docked with the corresponding microorganisms’ receptors. The results depict that compound **5d** shows comparable biological activities to these of the standard antibacterial/antifungal drugs in case of *S. aureus* and *C. albicans*. In addition, compound **9d** demonstrated the highest activity against *P. aeruginosa*.

## Figures and Tables

**Table 1:** MIC of the synthesized compounds against sensitive and resistant microorganisms.

Sample	Minimum Inhibitory Concentration (µg/mL)					
	Gram Positive Bacteria		Gram Negative Bacteria		Fungus	
	Sensitive *Staphylococcus aureus* ATCC 25923	Methicillin-Resistant *Staphylococcus aureus* (MRSA) ATCC-BAA-1720	Sensitive *Pseudomonas aeruginosa* ATCC10145	Penicillins and Cephalosporins-Resistant *Pseudomonas aeruginosa* ATCC BAA-2108	Azole-Sensitive *Candida albicans* ATCC 18804	Azole-Resistant *Candida albicans* ATCC 10231
**5a**	1.91	3.82	18.23	1.55	NA ^a^	NA
**5b**	3.37	1.85	3.92	14.29	2.61	NA
**5c**	7.81	5.74	NA	NA	62.5	NA
**5d**	0.15	0.49	NA	NA	0.29	19.07
**5e**	1.82	2.91	5.07	62.5	25.48	NA
**5f**	31.25	15.13	3.04	1.79	22.93	NA
**9a**	1.90	5.30	3.97	14.81	3.93	13.91
**9b**	5.29	1.27	10.13	NA	11.53	NA
**9c**	0.43	3.13	NA	NA	NA	NA
**9d**	0.71	0.42	0.93	1.84	0.65	2.17
**9e**	0.48	9.19	0.93	3.91	4.49	15.04
**13**	9.04	7.29	NA	NA	250	NA
**14**	0.49	1.13	NA	1.49	0.18	3.18
Vancomycin	0.24	0.98	0.49	3.9	ND	ND
Amphotericin B ^b^	ND	ND	ND	ND	0.24	1.95

^a^ NA, not active ^b^ ND, not determined.

**Table 2:** Quantum chemical parameters of compounds **6d, 6e, 10c** and **10d**.

Parameter	5d	5e	9c	9d
E_t_ (eV)	−40.188	−49.578	−50.556	−63.062
E_LUMO_ (eV)	−3.13	−3.43	−3.65	−3.72
E_HOMO_ (eV)	−6.01	−6.47	−6.58	−6.77
ΔE (eV)	2.87	3.03	2.93	3.06
Ionization energy (eV)	6.01	6.47	6.58	6.77
Electron affinity (eV)	3.13	3.43	3.65	3.72
Mulliken electronegativity	4.57	4.95	5.11	5.25
Softness	0.695	0.659	0.683	0.654
Hardness	1.437	1.517	1.464	1.529
Chemical potential (eV/mol)	−4.57	−4.95	−5.11	−5.25
Electrophilicity index	7.27	8.07	8.93	9.00

## Data Availability

The data presented in this study are available on request from corresponding author.

## Sample Availability

Samples of all synthesized compounds are available from the authors.
